# Detection of Infectivity in Blood of Persons with Variant and Sporadic Creutzfeldt-Jakob Disease

**DOI:** 10.3201/eid2001.130353

**Published:** 2014-01

**Authors:** Jean Yves Douet, Saima Zafar, Armand Perret-Liaudet, Caroline Lacroux, Séverine Lugan, Naima Aron, Herve Cassard, Claudia Ponto, Fabien Corbière, Juan Maria Torres, Inga Zerr, Olivier Andreoletti

**Affiliations:** Institut National de la Recherche Agronomique/Ecole Nationale Vétérinaire, Toulouse, France (J.Y. Douet, C. Lacroux, S. Lugan, N. Aron, H. Cassard, F. Corbière, O. Andréoletti);; National Reference Center for Transmissible Spongiform Encephalopathy, Georg August University, Göttingen, Germany (S. Zafar, C. Ponto, I. Zerr);; Hospices Civils de Lyon, France (A. Perret-Liaudet);; BioRan, Bron, France (A. Perret-Liaudet);; Centro de Investigación en Sanidad Animal, Madrid, Spain (J.M. Torres)

**Keywords:** Creutzfeldt-Jakob disease, CJD, vCJD, sCJD, prions, blood, infectivity, transmissible spongiform encephalopathy, TSE

## Abstract

We report the presence of infectivity in erythrocytes, leukocytes, and plasma of 1 person with variant Creutzfeldt-Jakob disease and in the plasma of 2 in 4 persons whose tests were positive for sporadic Creutzfeldt-Jakob disease. The measured infectivity levels were comparable to those reported in various animals with transmissible spongiform encephalopathies.

Among humans, Creutzfeldt-Jakob disease (CJD) is a low incidence disease (≈1 case per million per year) that occurs as either a sporadic (sCJD) or a familial/genetic (fCJD) form. Whereas familial disease forms are linked to a mutation in the prion protein gene (*Prnp*), no clear epidemiologic risk factors have been identified for sporadic disease forms. sCJD is not a uniform disorder in terms of clinical and neuropathological phenotype. sCJD cases are classified as type 1 or 2 according to the polymorphism at codon 129 of the protease-resistant prion protein (PrP) sequence (methionine/valine) and to the electromobility of the proteinase K–resistant core of the abnormal PrP (PrP^res^) (*1*). Type 1 and type 2 isoforms in sCJD are believed to correspond to different transmissible spongiform encephalopathy (TSE) agents

Despite their relative rarity, several hundred iatrogenically transmitted CJD cases were identified during the past 60 years (*2*). Some data supporting the presence of infectivity in the blood of sCJD-affected patients were reported following the intracerebral inoculation of blood fractions from affected patients into rodents. These observations remain ambiguous because other studies did not confirm them (*3*,*4*).

In 1996, a new form of CJD, named variant CJD (vCJD), was identified in humans. Variant CJD was demonstrated to be caused by the agent that causes bovine spongiform encephalopathy in cattle (*5*). In the United Kingdom, 4 vCJD transmissions (3 clinical cases and 1 asymptomatic infection) were probably caused by the transfusion of non–leuco-depleted erythrocyte concentrates prepared from donors who later had positive test results for vCJD (*6*). More recently, a presumed additional case of vCJD infection was reported in the United Kingdom in a hemophilic patient who had received fractionated plasma products, including some units linked to a donor who had vCJD diagnosed with vCJD (*7*). Despite the epidemiologic evidence of bloodborne transmission in vCJD, bioassays performed on conventional rodent models failed to demonstrate the presence of infectivity in the blood (*8*). The lack of TSE transmission in conventional rodent models could be a consequence of a low infectivity level in blood from vCJD- and sCJD-affected patients (as described in sheep and rodent TSE models) (*9*) or of the existence of the species barrier phenomenon that limits the transmission of human prions to these animal models. The development during the last decade of transgenic mice models expressing PrP from others species that abrogate the species barrier now offers the potential to detect low level of infectivity (*10*).

In this study, we used 2 transgenic mouse models that displayed a high sensitivity to the vCJD or sCJD TSE agents to estimate the infectious titer in certain blood fractions from vCJD- and sCJD-affected patients. According to legislation of the United Kingdom, Germany, and France, the experimental protocol, including the use of human samples, was approved by UK National CJD Research & Surveillance Unit tissue bank: REC reference number 2000/4/157-German TSE reference center: Ref Nr 11/11/93, PHRC ref 2004-D50-353 for patient from France.

## The Study

Previous studies reported a high sensitivity in transgenic mice overexpressing bovine PrP (tgBov) for the detection of the bovine spongiform encephalopathy agent. To demonstrate that tgBov also displays a high sensitivity to vCJD infection, we titrated to endpoint a vCJD isolate (10% brain homogenate) by intracerebral inoculation in this model (Tg110) (*11*). Considering the potential diversity of TSE agents that may cause sCJD, we decided to focus only on type 1 homozygous for methionine at codon 129 of the PRP gene (MM1) sCJD cases. An endpoint titration of a MM1 sCJD 10% brain homogenate was performed in a mouse model that express the methionine 129 variant of the human PrP gene (tgHu:Tg340) (*12*). This enabled confirmation of the capacity of the tgBov and tgHu models to detect the vCJD and sCJD MM1 agent, respectively, up to a 10^−6^ dilution of the reference brain homogenates (Table 1; *13*). This value was within the range of the brain/blood relative infectivity reported in various TSE animal models (*9*,*14*).

**Table 1:** Titration of sCJD and vCJD isolates in transgenic mice expressing the human or bovine prion protein*†

Dilution	sCJD MM1 in tgHu			vCJD in tgBov	
	Positive transmission in mice	Incubation period, d		Positive transmission in mice	Incubation period, d
Not diluted	6/6	186 ± 10		6/6	249 ± 2
10^−1^	6/6	213 ± 15		6/6	283 ± 15
10^−2^	6/6	240 ± 13		6/6	316 ± 21
10^−3^	6/6	263 ± 24		6/6	342 ± 10
10^-4-^	6/6	296 ± 26		6/6	453 ± 66
10^−5^	6/6	323 ± 29		4/6	499 ± 17
10^−6^	1/6	316		1/6	502
10^−7^	0/6	>650		0/6	>700
Infectious titer, ID_50_/g of brain (95% CI)	10^6.67^ (10^6.33^−10^6.97^)			10^6.33^ (10^5,84^ −10^6.82^)	

*sCJD, sporadic Creutzfeld-Jakob Disease; tgHu, human PrP gene ;PrP, protease-resistant prion protein; vCJD, variant CJD; tgBov transgenic mice overexpressing bovine PrP, ID, infectious dose. †Successive 1/10 dilutions of 10% brain homogenate (frontal cortex) from patients affected by vCJD and sCJD were injected intracerebrally to tgHu (n = 6) and tgBov (n = 6) mice, respectively. Those 2 patients were different from the 1 whose blood was tested in bioassay (Table 2). Mice were euthanized when they showed clinical signs of infection or after 650 days postinfection. Mice were considered infected when abnormal prion protein deposition was detected in the brain by western blot by using Sha31 monoclonal antibody, which recognizes amino acids 145–152 (YEDRYYRE) of the sheep prion protein. Infectious titers were estimated by the Spearman-Karber method (*14*).

In the next step of our experiment, blood fractions (erythrocytes, plasma, and leukocytes) from 1 vCJD-confirmed patient were injected intracerebrally in tgBov mice. Similarly, plasma samples from 4 sCJD MM1 patients were inoculated with tgHu (Table 2). The blood fraction preparation was performed by using laboratory scale hematologic protocols (Technical Appendix), not by following the procedure applied by blood banking services. This method implies that the leucodepletion that is applied to blood labile products in most countries to reduce the vCJD bloodborne transmission risk was not performed. Brain tissue samples from each of the 4 sCJD cases were also inoculated with tgHu. On the basis of the incubation period (Table 2) and PrP^res^ distribution pattern in the brain as assessed by using paraffin-embedded tissue blot, the TSE agents in those isolates were indistinguishable from those in the MM1 sCJD case that was used for endpoint titration (Figure, panel A).

**Table 2:** Intracerebral inoculation of blood components collected from 1 vCJD and 4 sCJD cases (MM1) in transgenic mice expressing the bovine or human prion protein gene*†

Mouse model	Donor	Specimen	Inoculated mice	Positive mice	Incubation period, d	ID/mL (95%CI)‡
tgBov	vCJD	Leukocyte	24	3	476, 567, 576	2.23 (0–4.87)
		Plasma	24	1	453	2.12 (0–6.52)
		Erythrocyte	24	1	433	2.12 (0–6.52)
tgHu	sCJD case 1	Plasma	14§	1	338	3.70 (0–11.65)
		Brain	6	6	216 ± 2	NA
	sCJD case 2	Plasma	24	0	>700	0 (0- 6.24)
		brain	6	6	217 ± 5	NA
	sCJD case 3	Plasma	24	1	233	2.12 (0–6.52)
		Brain	6	6	205 ± 5	NA
	sCJD case 4	Plasma	24	0	>700	0 (0–6.24)
		Brain	6	6	207 ± 3	NA
tgHu	Control human	Plasma	12	0	>650	NA
tgBov	Control human	Plasma	12	0	>650	NA
tgHu	Control human	PBS	12	0	>700	NA
tgBov	Control human	PBS	12	0	>700	NA
tgHu	Control human	Brain	24	0	>700	NA
tgBov	Control human	Brain	24	0	>700	NA
tgHu	Control human	None	24	0	>750	NA
tgBov	Control human	None	24	0	>750	NA

*vCJD, variant Creutzfeld-Jakob disease; sCJD, sporadic Creutzfeld-Jakob disease; dpi, days postinfection; ID, infectious dose; tgBov, bovine prion protein; tgHu, human prion protein;; PBS, phosphate-buffered saline.  †The leukocyte(s) from a single vCJD case corresponding to a starting volume of 3 mL of blood were suspended in 1 mL of 5% glucose solution. The leukocyte suspension and the crude erythrocytes were homogenized by using a high speed cell disrupter. The leukocyte and erythrocyte homogenates (vCJD case) and crude plasma (vCJD and sCJD cases) were intracerebrally injected into mice (20 µL per mouse). For the 4 sCJD MM1 cases, brain homogenate (10%, temporal cortex) were also inoculated in tgHu. Mice were euthanized when they showed clinical signs of infection or after 650 or 750 dpi. Mice were considered infected when abnormal protease-resistant prion protein; deposition was detected in brain tissue by using Western blot analysis with Sha31 monoclonal antibody: epitope amino acids 145–152 (YEDRYYRE) of the sheep PrP sequence. For samples showing 100% attack rate, incubation periods are reported as mean (± SD). For other samples, individual incubation period of CJD-positive mice are presented; their infectious titers were estimated by using limiting dilution titration method (application of Poisson model) described by Brown et al (*13*). ‡Leukocyte titer is expressed as ID/mL of the starting whole blood. Plasma and erythrocyte titers are expressed as ID/mL of inoculum. §24 mice were inoculated; 10 died because of the acute toxicity of the sample.

**Figure:**
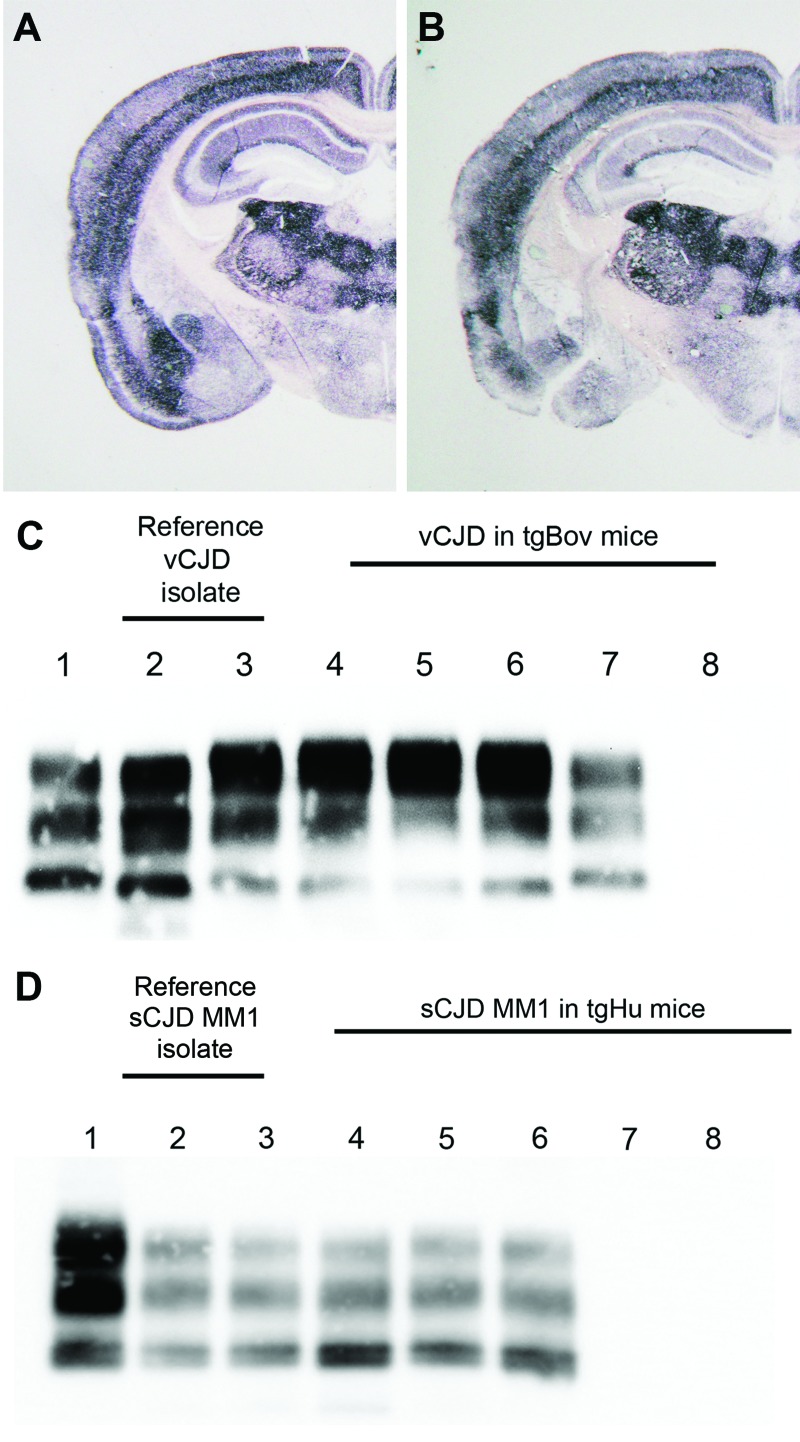
Abnormal prion protein (PrP^res^) detection by using Western blot (WB) and paraffin-embedded tissue (PET) blot in the brain of transgenic mice expressing the methionine 129 variant of the human prion protein (PrP) (tgHu) or bovine PrP (tgBov). A, B) PET blot PrP^res^ distribution in coronal section (thalamus level) of tgHu mice inoculated with sporadic Creutzfeldt-Jakob disease (sCJD) MM1 isolates (10% brain homogenate): A) reference isolate used for the endpoint titration in Table 1; B) sCJD case 1 (Table 2). C) PrP^res^ WB of variant Creutzfeldt-Jakob disease (vCJD) reference isolate (used for endpoint titration in Table 1) and tgBov mice inoculated with the same vCJD reference isolate or vCJD blood fractions. Lane 1, WB-positive control; lanes 2 and 3, reference vCJD isolate; lane 4, leukocytes; lane 5, erythrocytes; lane 6, plasma; lane 7, WB-positive control; lane 8, healthy human plasma in tgBov. D) PrP^res^ Western blot of the sCJD reference isolate (used for endpoint titration in Table 1) and tgHu mice inoculated with the same sCJD reference isolate and plasma from sCJD cases. A proteinase K–digested classical scrapie isolate in sheep was used as positive control for the blots in panels C and D. PrP^res^ immunodetection in PET and Western blots was performed by using Sha31 monoclonal antibody (epitope: _145_YEDRYYRE_152_ of the human PrP). Lane 1, WB-positive control; lanes 2 and 3, reference sCJD MM1 isolate; lane 4, brain tissue from case 1; lane 5, plasma from case 1; lane 6, plasma from case 3; lane 7, plasma from case 2; lane 8, plasma from case 4.

No TSE clinical signs or PrP^res^ accumulation were observed in the tgBov or tgHu mice inoculated with phosphate-buffered saline or brain and plasma from healthy human controls. The 3 blood fractions from the vCJD-affected patient caused a positive result but low attack rate among tgBov mice (Table 2). On the basis of these results, infectivity in erythrocytes and plasma was estimated to be 2.12 infectious dose (ID)/mL of inoculum. In leukocytes, the infectious titer was estimated to be 2.23 ID/mL of whole blood. According to these values and the hematocrit of the sample (Technical Appendix), the global infectious titer whole blood in the tested patient would be ≈4.45 ID/mL. Such infectious level is approximately equivalent to 1.4 µg of the reference vCJD brain sample that was endpoint-titrated (Table 1).

In tgHu mice, positive transmission was observed among mice inoculated with 2 of 4 plasma samples (Table 2). The infectious titers in both positive plasma samples were estimated to be 2.12 and 3.7 ID/mL of plasma, which is equivalent to 0.3–0.5 µg of the reference sCJD MM1 brain sample that was endpoint titrated (Table 1). However, because of the limited number of mice inoculated (n = 24) and the overall sensitivity of the assay (upper CI limit 6.24 ID/mL), the absence of transmission in mice inoculated with the 2 other plasma samples cannot be interpreted conclusively

In tgBov inoculated with vCJD and tgHu inoculated with sCJD, the PrP^res^ banding patterns observed by Western blot in animals challenged with brain homogenate and blood components were identical (Figure, panels C, D). These results support the contention that the TSE agent propagated in tgBov mice and tgHu were vCJD and sCJD agents, respectively.

## Conclusions

The data reported here confirm the presence of infectivity in erythrocytes, leukocytes, and plasma from vCJD-affected patients and demonstrate unambiguously the presence of infectivity in the plasma of some, but not all, sCJD-affected patients. The infectivity levels that we measured in the tested vCJD and sCJD blood components were comparable to those reported in various TSE animal models. The number of cases included in our study was limited; a new experiment that would include a larger number of cases and different blood fractions from sCJD cases will be necessary to refine the data. However, these results represent a substantial input for assessing the risk for interindividual bloodborne transmission of sCJD and vCJD.

Technical Appendix Biochemical typing and PrP ORF sequencing of sporadic and variant Creutzfeldt-Jakob disease genes.
