# Supplementary material for: Detection of Infectivity in Blood of Persons with Variant and Sporadic Creutzfeldt-Jakob Disease
Source: Emerg Infect Dis. 2014 Jan;20(1):114–7. doi: 10.3201/eid2001.130353 (PMC3884712; doi:10.3201/eid2001.130353)
Supplement: Technical Appendix — Biochemical typing and PrP ORF sequencing of sporadic and variant Creutzfeldt-Jakob disease genes. [file 13-0353-Techapp-s1.pdf]

Article DOI: http://dx.doi.org/10.3201/eid2001.130353

Detection of Infectivity in Blood of Persons with Variant and Sporadic Creutzfeldt-Jakob Disease

Technical Appendix

Biochemical Typing and PrP ORF Sequencing of Sporadic and Variant Creutzfeldt-Jakob Disease Genes

Confirmation of the disease diagnosis, PrP^{res} WB typing and *PrnP* gene sequencing in the patients were performed by the national CJD reference center of the country of origin of each patient. All patients were Methionine/Methionine at codon 129 and no other mutation was observed. sCJD cases were all originating from Germany. The vCJD case whose blood was tested by bioassay was originating from France. The vCJD case that was used in the endpoint titration experiment was provided by the UK CJD reference center in Edinburgh.

Blood Collection and Fractionation

sCJD blood samples were collected by using S-Monovette® Coagulation Sodium Citrate 1 in 3 mL tubes according to manufacturer instruction (SARSTEDT AG & Co. · www.sarstedt.com) . Tubes were centrifuged for 20 minutes at 2000 rpm, plasma was then collected and cell-free fraction underwent another centrifugation step at 13000 rpm for 10 minutes. Supernatant was collected and stored frozen. The hematocrit values corresponding to the different samples were: sCJD case 1: 37.6%, sCJD case 2: 39.7%, sCJD case 3: 43%, sCJD case 4: 43.7%.

vCJD blood sample on EDTA and fractionated by a 10 minutes 3000 g centrifugation at 12°C . Plasma was collected and directly frozen stored. The buffy coat was collected and washed twice in NaCl 0.9% (2 min, RT) before being pelleted at 3000 g 10 min and frozen.

The sample was submitted to standard biochemical analyze and the blood formula was red cells 5.21 10^{12}/L, hemoglobin 149 g/L, hematocrit: 48%, total white cells 17.1 10^{9}/L, lymphocytes: 27.1%, monocytes 9.3%, neutrophils: 60%, eosinophil: 1.8%, Basophils: 1.8%, Platelets:356 10^{9}/L.

Brain and Blood Samples Handling and Bioassay

Blood was collected during the diagnostic procedures when patients were evaluated for CJD diagnosis at notifying hospital. The time between blood sampling and patients' decease are reported in Technical Appendix Table 1.

For sCJD patients, blood was processed at the CSF reference laboratory of the National TSE Reference Center at the Department of Neurology Göttingen, Germany. Autopsy was performed by the Department of Pathology of the notifying hospital and reference material was sent to the Department of Neuropathology, Göttingen, Germany. Blood and brain samples were stored in separate department and handled by different staff in the Gottingen University hospital.

The vCJD blood sample was collected and fractionated in the Bron Hospital (France). In this hospital the department handling CSF and blood samples and the pathology department (post mortem sampling) are distinct. The vCJD reference brain sample was obtained from the UK CJD reference laboratory in Edinburgh.

All the samples were dispatched to the laboratory that performed the bioassays (UMR INRA ENVT 1225) in separated sealed containers. Samples were kept untouched and prepared only a few hours before their inoculation in mice.

The sCJD endpoint titration in tgHu mice was performed 1 year before the reception of sCJD plasma samples.

Plasma and Brain samples from the four sCJD affected patients were prepared and inoculated separately; Brain from the affected patients (text Table 2) were inoculated after the first positive transmission occurred in mice inoculated with sCJD plasma.

Similarly the vCJD endpoint titration experiment and the inoculation of the vCJD blood samples in tg Bov were performed at different dates (9 months interval).

Negative control (phosphate-buffered saline and healthy blood samples) were inoculated during the same inoculation session than the inoculation of the blood fractions from the vCJD and sCJD patients. Healthy brain controls (human and bovine) were inoculated during the same session than the endpoint titration of sCJD and vCJD brain material.

Technical Appendix Table. Clinico-pathological data and medical history in variant and sporadic Creutzfeld-Jakob Disease patients whose blood samples were tested in bioassay*

Patient	Sex	Age at onset	Disease duration	Blood collection†	CSF 14–3-3	MRI	EEG	Medical and surgical history
vCJD	M	46	12	12	–	+	–	None
sCJD 1	F	75	8	7	+	–	+	Gynecological
sCJD 2	F	75	3	2	+	+	+	Gynecological, orthopedic, hip fracture and replacement
sCJD 3	F	68	13	5	+	+	+	Cataract, hip replacement, tonsillectomy
sCJD 4	M	66	2	2	+	–	+	Hernia, appendectomy, tonsillectomy

*vCJD, variant Creutzfeld-Jakob Disease; sCJD. sporadic Creutzfeld-Jakob Disease; CSF, cerebrospinal fluid. EEG, electroencephalogram; MRI, Magnetic resonance imaging.
†Number of months after disease onset.
